# Time-Dependent miRNA Profile during Septic Acute Kidney Injury in Mice

**DOI:** 10.3390/ijms21155316

**Published:** 2020-07-27

**Authors:** Pál Tod, Beáta Róka, Tamás Kaucsár, Krisztina Szatmári, Matej Vizovišek, Robert Vidmar, Marko Fonovič, Gábor Szénási, Péter Hamar

**Affiliations:** 1 Institute of Translational Medicine, Semmelweis University, 1094 Budapest, Hungary; todpal90@gmail.com (P.T.); beata.roka@gmail.com (B.R.); kaucsar.tamas@med.semmelweis-univ.hu (T.K.); szatmaritina@gmail.com (K.S.); szenasi.gabor@med.semmelweis-univ.hu (G.S.); 2 Institute for Translational Medicine, Medical School, University of Pécs, 7624 Pécs, Hungary; 3 Department of Biochemistry and Molecular and Structural Biology, Jožef Stefan Institute, 1000 Ljubljana, Slovenia; vizovisek@imsb.biol.ethz.ch (M.V.); Robert.Vidmar@ijs.si (R.V.); marko.fonovic@ijs.si (M.F.); 4 Centre of Excellence for Integrated Approaches in Chemistry and Biology of Proteins, 1000 Ljubljana, Slovenia

**Keywords:** septic acute kidney injury, miArray, miR-21a, miR-144-3p, miR-146a-5p, miR-451a, miR-762, aquaporin-1, cross-tolerance

## Abstract

(1) Background: Lipopolysaccharide (LPS)-induced systemic inflammation is associated with septic acute kidney injury (AKI). We investigated the time-dependent miRNA expression changes in the kidney caused by LPS. (2) Methods: Male outbred NMRI mice were injected with LPS and sacrificed at 1.5 and 6 h (40 mg/kg i.p., early phase, EP) or at 24 and 48 h (10 mg/kg i.p., late phase, LP). The miRNA profile was established using miRCURY LNA™ microarray and confirmed with qPCR. Total renal proteome was analyzed by LC-MS/MS (ProteomeXchange: PXD014664). (3) Results: Septic AKI was confirmed by increases in plasma urea concentration and in renal TNF-α and IL-6 mRNA expression. Most miRNAs were altered at 6 and 24 h and declined by 48 h. In EP miR-762 was newly identified and validated and was the most elevated miRNA. The predicted target of miR-762, Ras related GTPase 1B (Sar1b) was downregulated. In LP miR-21a-5p was the most influenced miRNA followed by miR-451a, miR-144-3p, and miR-146a-5p. Among the potential protein targets of the most influenced miRNAs, only aquaporin-1, a target of miR-144-3p was downregulated at 24 h. (4) Conclusion: Besides already known miRNAs, septic AKI upregulated miR-762, which may regulate GTP signaling, and miR-144-3p and downregulated its target, aquaporin-1.

## 1. Introduction

The estimated incidence of acute kidney injury (AKI) in hospital admissions is 8–17% [1]. The main causes of AKI are ischemia-reperfusion injury and septic shock [2]. Septic AKI is common among the critically ill with an increasing incidence and increasing risk of death [3]. Lipopolysaccharide (LPS), a component of the Gram-negative bacterial cell wall, is a potent and well-characterized immunostimulant [4]. LPS administration induces a systemic inflammatory response, and increases the expression of pro-inflammatory cytokines such as TNF-α and IL-6 [5]. Depending on the dose, LPS can cause AKI in various animal species [6,7,8,9].

MicroRNAs are powerful modulators of posttranscriptional gene expression and influence pathologic processes such as septic AKI. There are few miRNAs associated previously with LPS effects in the kidney. The role of the best-characterized miR-223 seems to be controversial. MiR-223 was demonstrated to exaggerate a sterile model of septic AKI (LPS), while it attenuated polymicrobial infection-induced AKI in miR-223 KO mice [10]. Furthermore, the model-specific role of miR-223 can be organ-dependent as myocardial dysfunction and mortality was aggravated in polymicrobial infection in miR-223 KO mice [11] contrary to kidney injury. The results highlight the organ-specific effects of miRNAs, which can depend on the functional status of the cells and the activity of many other regulatory factors involved in the potentially protective or harmful effects.

In the present study, we evaluated miRNA expression changes in the kidney during LPS-induced AKI. Although, there are several reports on the role of miRNAs in LPS-induced inflammation [12,13,14,15], to our best knowledge, this is the first miRNA microarray investigating the miRNA profile changes in septic AKI. Deeper understanding of the miRNA machinery of the kidney may lead to therapeutic advances in septic AKI.

## 2. Results

### 2.1. LPS-Induced Renal Pro-Inflammatory Cytokine Production

**Figure 1:**
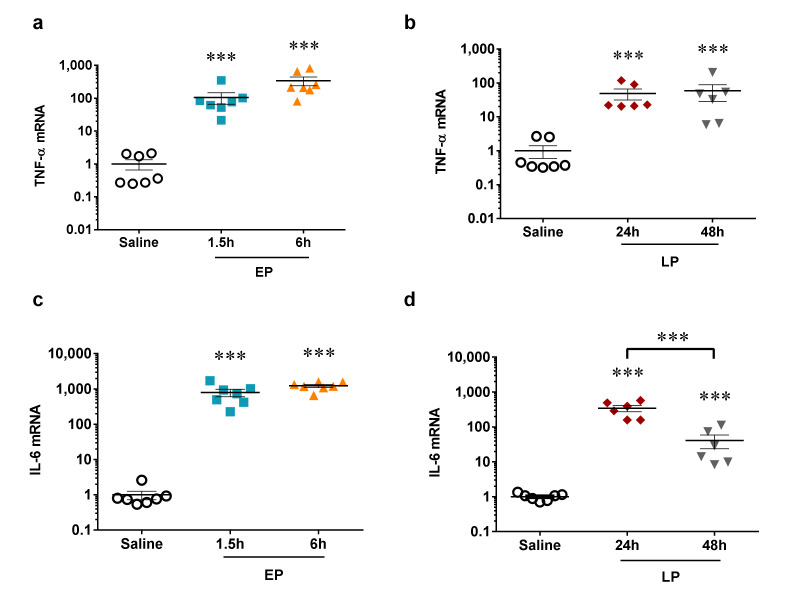
The relative mRNA expression of pro-inflammatory proteins after lipopolysaccharide (LPS) injection normalized to GAPDH (fold changes vs. saline). (**a**,**b**) TNF-α mRNA (**a**) early phase (EP), 1.5 and 6 h, (**b**) late phase (LP), 24 and 48 h, (**c**,**d**) IL-6 mRNA (**c**) EP, 1.5 and 6 h, (**d**) LP, 24 and 48 h. Data are expressed as mean ± SEM; one-way ANOVA; ***: *p* < 0.001.

LPS administration induced two orders of magnitude increases in pro-inflammatory cytokine (TNF-α and IL-6) mRNA in the kidneys that was similar in the early and late phases of AKI and independent of the dose and time (Figure 1). IL-6 but not TNF-α started to reverse 48 h after LPS administration.

### 2.2. LPS-Induced Reversible Acute Kidney Injury

**Figure 2:**
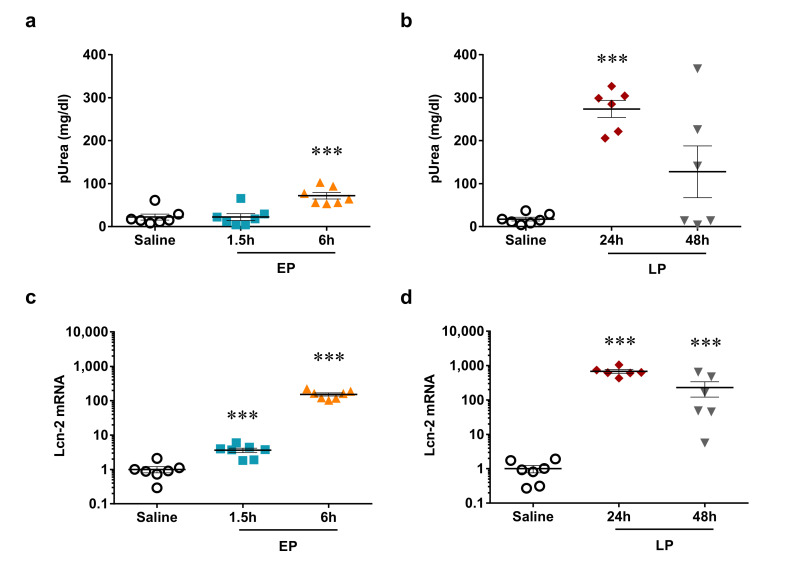
Acute kidney injury markers. (**a**,**b**) Plasma urea concentration after LPS injection, (**a**) EP, 1.5 and 6 h, (**b**) LP, 24 and 48 h; (**c**,**d**) Lcn-2 mRNA normalized to GAPDH (fold changes vs. saline), (**c**) EP, 1.5 and 6 h, (**d**) LP, 24 and 48 h. Data are expressed as mean ± SEM; one-way ANOVA; ***: *p* < 0.001.

The renal retention parameter, plasma urea concentration was elevated first at 6 h after LPS administration, and increased further at 24 h despite a lower LPS dose. Renal damage started to reverse 48 h after LPS as plasma urea concentration was lower at 48 h vs. 24 h (Figure 2a,b).

The tubular injury marker, renal lipocalin-2 (Lcn-2, also named: neutrophil gelatinase-associated lipocalin, NGAL) mRNA was upregulated already at 1.5 h, and increased further at 6 h after LPS administration. Lcn-2 mRNA was similarly elevated at 6 and 24 h, but just as plasma urea and IL-6, it began to decline at 48 h (Figure 2c,d). 

Taken together, kidney injury was most severe 24 h after LPS administration and recovery started at 48 h.

### 2.3. miRNA Array Profiling Revealed Three Differently Expressed Clusters

**Figure 3:**
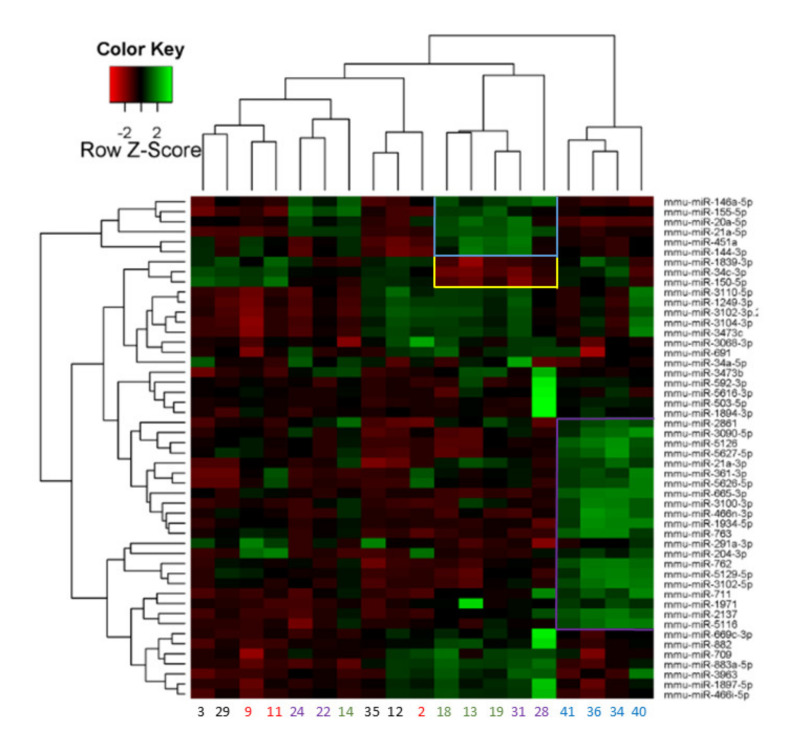
Two-way hierarchical clustering of the top 50 miRNAs with the largest difference between LPS and saline-treated mouse kidneys. Each row represents a miRNA and each column represents a sample. Clustered miRNAs are marked with blue-, yellow-, and purple frames. Samples are saline: #3, 29, 35; 12, EP: 1.5 h: #9, 11, 2; 6 h: #41, 36, 34, 40; LP: 24 h: #14, 18, 13, 19; 48 h: #24, 22, 31, 28 (based on the heat-map provided by Exiqon).

In order to study differentially expressed miRNAs after LPS administration 1195 miRNAs were analyzed by microarray (Figure 3). Based on the Lowess regression analysis, one sample was excluded as an outlier and 862 miRNAs were excluded because they were undetectable in more than 90% of the samples. From the remaining 333 miRNAs, 71 miRNAs were significantly altered in EP and 39 in LP (Figure 3). MiRNAs with a fold change above 1.5 or below 0.75 (>150% or <75%) were considered biologically important and studied further.

The miRNA microarray showed no distinct clusters at 1.5 h, thus control and 1.5 h samples are all on the left side of the heat-map. However, 21 miRNAs (Cluster I, purple frame) were jointly downregulated at 6 h on the right side of the heat map. Thus, all four animals in the 6-h group were clustered together. Other clusters were identified at LP with six upregulated (Cluster II, blue frame) and three downregulated miRNAs (Cluster III, yellow frame) (Table 1). Mice in the LP groups underexpressing cluster III and overexpressing cluster II were mostly from the 24-h group (Nr. 13, 18, 19) whereas Nr. 31 was from the 48-h group. Based on the much stronger upregulation of several miRNAs animal Nr. 28 seems to be an outlier. MiRNA expression changes in the other mice belonging to the 48-h group (Nr. 22, 24) seem to be less intense, therefore these mice were clustered with mice in the control and 1.5-h groups. This suggests a decline in miRNA response 48 h after LPS.

Microarray results were further analyzed by ranking differentially expressed miRNAs based on their fold changes. One miRNA was elevated at 1.5 h and 15 miRNAs were upregulated at least 1.5 times at 6 h compared to the control group (Table 2). Thus, miRNA expression changes practically started at 6 h. No miRNA was significantly downregulated in the early phase (EP) groups with a fold change below 0.75.

In the late phase (LP) six miRNAs were significantly elevated at least 1.5× and five were decreased at 24 h. At 48 h only miR-21a-5p and miR-146a-5p increased and five miRNAs decreased (Table 2). Thus, most miRNAs affected at 24 h did not change at 48 h, supporting the observation based on the heat-map clustering that miRNA expression changes were milder at 48 h. Most of the miRNAs in both EP and LP groups were also identified by the heat-map cluster analysis (grey highlights in Table 1 and Table 2). Thus, results of the cluster-analysis were supported by the analysis of ranks. Taken together, most miRNAs were upregulated at the time of the peak inflammatory reactions i.e., at the peak of the renal injury at 6 (15 miRNAs) and 24 h (11 miRNAs).

### 2.4. miRNA Microarray Validation

**Figure 4:**
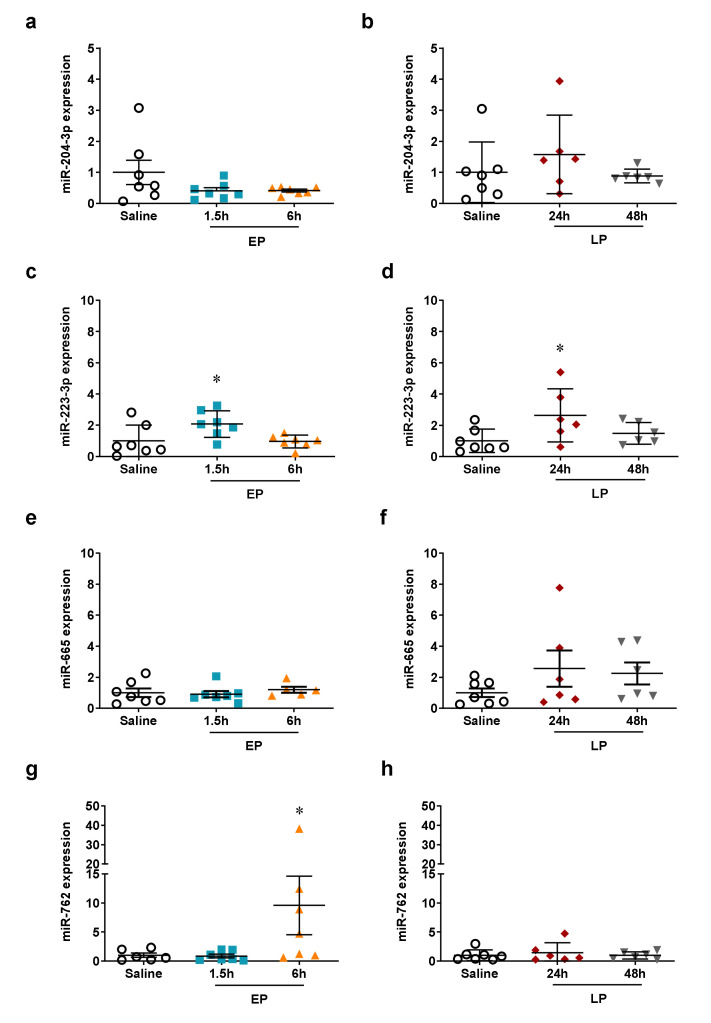
qPCR validation of miRNAs upregulated during the early phase on the microarray. The relative expression of miRNAs normalized to let-7g-5p (fold changes vs. saline). Left column: EP, right column: LP. (**a**,**b**) miR-204-3p; (**c**,**d**) miR-223-3p; (**e**,**f**) miR-665; (**g**,**h**) miR-762. Data are expressed as mean ± SEM; one-way ANOVA; *: *p* < 0.05.

The top four ranked miRNAs in each group were validated by qPCR in all samples. Expression changes of miR-762 were successfully validated at all time-points (Figure 4g,h). The changes in miR-204-3p and miR-665 expression at 1.5 and 6 h could not be confirmed (Figure 4a,b,e,f). The qPCR reaction did not yield any qPCR product in the cases of miR-2137 and -3102-5p. Furthermore, miR-223-3p was also measured by qPCR as several publications suggest its involvement in LPS-induced AKI. In our study, miR-223-3p was 15th in the miRNA microarray rank with a 1.6-fold increase at 6 h and a small but significant 1.3-fold increase at 24 h. The qPCR results demonstrated a significant increase at 1.5 and 24 h (Figure 4c,d).

**Figure 5:**
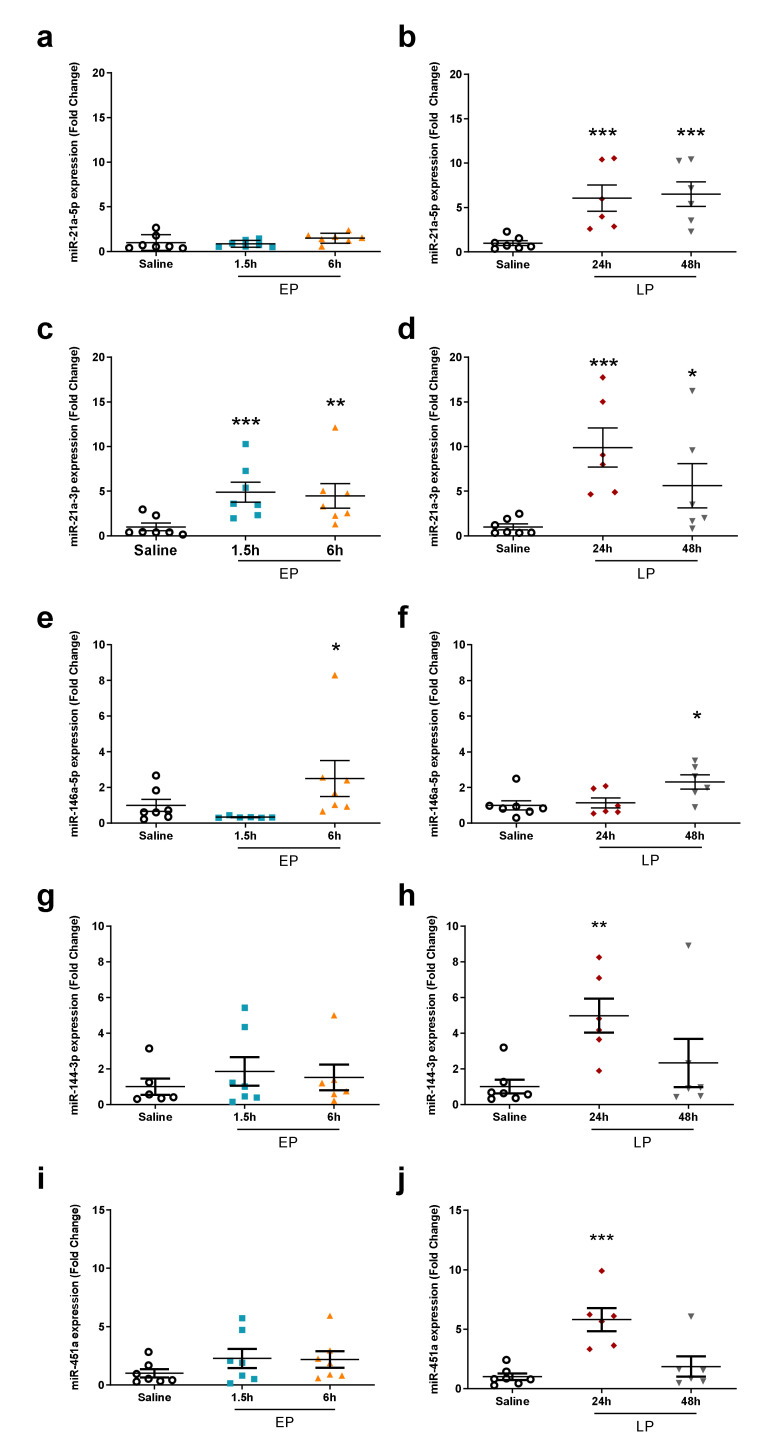
qPCR validation of miRNAs upregulated during the late phase on the microarray. The relative expression of miRNAs was normalized to let-7g-5p (fold changes vs. saline). Left column: EP, right column: LP. (**a**,**b**) miR-21a-5p; (**c**,**d**) miR-21a-3p; (**e**,**f**) miR-146a-5p; (**g**,**h**) miR-144a-3p; (**i**,**j**) miR-451a. Data are expressed as mean ± SEM; one-way ANOVA; *: *p* < 0.05, **: *p* < 0.01, ***: *p* < 0.001.

Increases in miR-21a-5p and miR-21a-3p expression were validated by qPCR at most time points (Figure 5a–d). The increased expression of miR-144-3p and miR-451a was also verified by qPCR at 24 h (Figure 5g–j). The elevated expression of miR-146a-5p was validated by qPCR only at 48 h, and a significant elevation was also seen at 6 h in contrast to the microarray results (Figure 5e,f).

**Figure 6:**
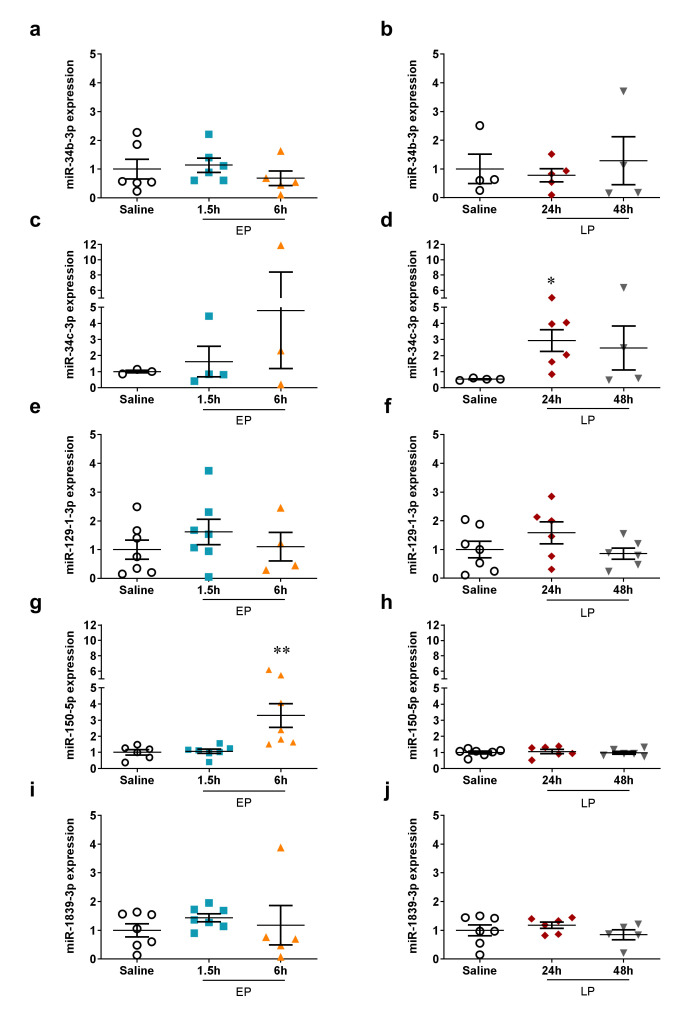
qPCR validation of miRNAs downregulated on the microarray. The relative expression of miRNAs normalized to let-7g-5p (fold changes vs. saline). Left column: EP, right column: LP. (**a**,**b**) miR-34b-3p; (**c**,**d**) miR-34c-3p; (**e**,**f**) miR-129-1-3p; (**g**,**h**) miR-150-5p; (**i**,**j**) miR-1839-3p. Data are expressed as mean ± SEM; one-way ANOVA; *: *p* < 0.05, **: *p* < 0.01.

None of the downregulated miRNAs in the miRNA microarray could be validated by qPCR (Figure 6). According to the PCR measurements miR-34b-3p, miR-129-1-3p, and miR-1839-3p expression was not altered by LPS. MiR-34c-3p and miR-150-5p were upregulated at 24 and 6 h, respectively, while they were unchanged at the other time points.

### 2.5. MicroRNA Targets Identified by Mass Spectrometry

**Figure 7:**
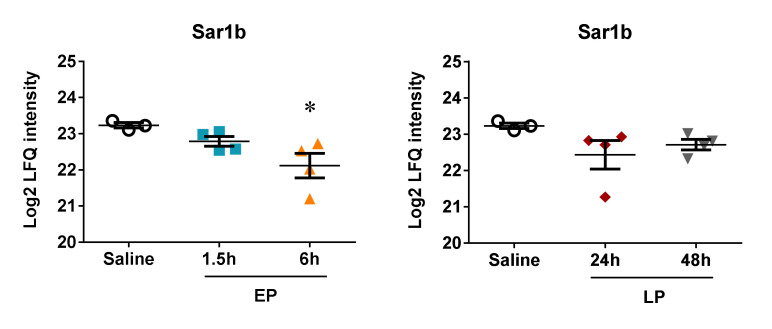
Protein expression of the miR-762 predicted target GTP-binding protein Sar1b in the kidneys of mice after LPS administration. Log2 transformed label-free quantification (LFQ) intensity values of proteins were determined by mass spectrometry. Left column: EP, right column: LP. Secretion associated Ras related GTPase 1B (Sar1b). Data are expressed as mean ± SEM; one-way ANOVA; *: *p* < 0.05.

Our MS analysis did not detect any of the so far experimentally validated targets of miR-762, the novel miRNA not associated with septic AKI or renal ischemia before. However, from the 732 predicted targets of miR-762 in the miR-data base (MiRDB), 31 were identified in most of the samples by the MS. One of these proteins demonstrated possibly relevant association with miR-762: the secretion associated Ras related GTPase 1B or GTP-binding protein Sar1b (Figure 7) was significantly reduced 6 h after LPS, at the time of the peak upregulation of miR-762.

**Figure 8:**
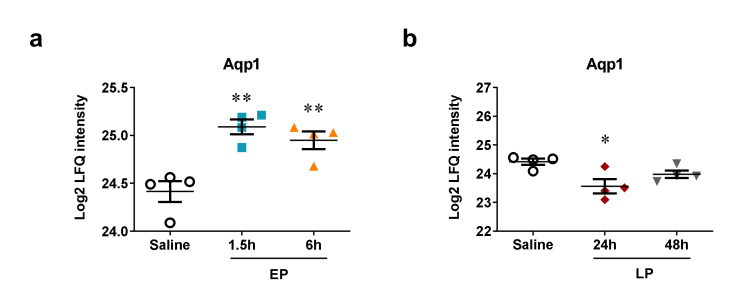
Protein expression of experimentally validated miRNA targets in the kidneys of mice after LPS administration. Log2 transformed label-free quantification (LFQ) intensity values of proteins as determined by mass spectrometry. Left column: EP, right column: LP. (**a**,**b**) Aquaporin-1 (Aqp1). Data are expressed as mean ± SEM; one-way ANOVA; *: *p* < 0.05, **: *p* < 0.01.

Another target of a differentially regulated miRNA detected by the MS was aquaporin-1 (Aqp1), a validated target of miR-144-3p. Aqp1 protein level significantly increased in EP and decreased at 24 h (Figure 8). In our study Aqp1 behaved inversely to miR-144-3p as it was upregulated (Table 1 Cluster II and Figure 5g,h) and Aqp1 was downregulated at 24 h after LPS.

Finally, the MS analysis revealed significantly changed transporter proteins, which are predicted or validated targets of the miRs identified by the miR-array and validated by qPCR.

**Figure 9:**
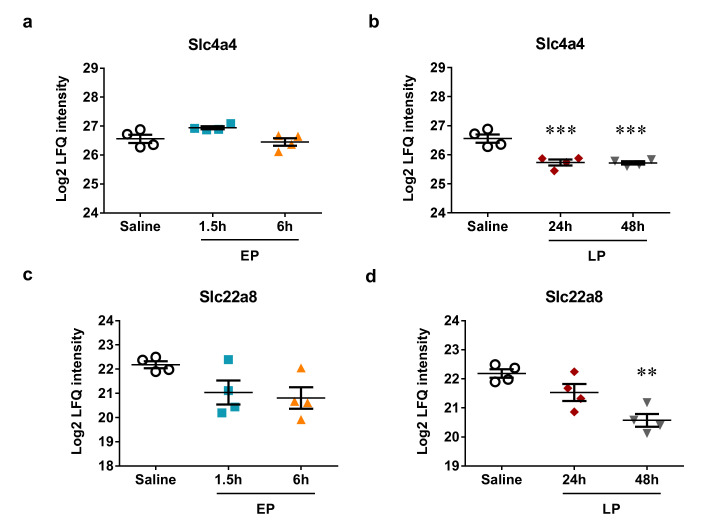
Protein expression of transporters that are predicted miRNA targets in mouse kidneys after LPS administration. Log2 transformed label-free quantification (LFQ) intensity values of proteins were determined by mass spectrometry. Left column: EP, right column: LP. (**a**,**b**) Slc4a4; (**c**,**d**) Slc22a8. Data are expressed as mean ± SEM; one-way ANOVA; **: *p* < 0.01, ***: *p* < 0.001.

The electrogenic sodium bicarbonate cotransporter 1 (NBCe1) or solute carrier family 4 member 4 (Slc4a4) is a predicted target of miR-223-3p and miR-144-3p. Slc4a4 was unchanged during the early phase, but was significantly downregulated at both time points during the late phase (Figure 9a,b). Both MiR-223-3p (Figure 4c,d) and miR-144-3p (Figure 5g,h) were upregulated most at 24 h.

Another transporter, solute carrier family 22 member 8 (Slc22a8) or organic anion transporter 3 (Oat3), a predicted target of miR-144-3p was also tendentially downregulated by LPS at all time points, but the downregulation was significant only 48 h after LPS (Figure 9c,d). Simultaneously, miR-144-3p was tendentially upregulated at all time-points by LPS, but the upregulation was significant only 24 h after LPS (Figure 5g,h).

## 3. Discussion

This is the first study investigating time-dependent changes in the renal miRNome following LPS administration. The miRNome responded to LPS injection mainly by upregulation with only a few downregulated miRNAs, which we could not confirm by qPCR. Despite severe renal functional impairment already at 1.5 h, as indicated by Lcn-2 mRNA, only one miRNA was upregulated at the same time. The most robust miRNA response to LPS was observed at 6 (15 miRNAs) and 24 (11 miRNAs) h. We observed a coordinated upregulation of a miRNA cluster at 6 h after LPS. Another clear expression pattern of six miRNAs was identified in the late phase. MiR-21a-duplex, miR-144-3p/451a, and miR-146a-5p were elevated at 24 or 48 h. The decline of urea and Lcn-2 at 48 h indicated recovery associated with a decline in the expression of upregulated miRNAs.

Several false positive hits were found among the microarray results upon validation by qPCR. According to the qPCR assays miR-204-3p and miR-665 (upregulated on the array) and miR-34b-3p and miR-1839-3p (downregulated on the array) were not influenced by LPS. Furthermore, miR-34c-3p significantly increased as determined by qPCR instead of a downregulation as suggested by the miRNA array. Finally, the qPCR reaction resulted in no amplification product for miR-2137 and 3102-5p. It is a long debate that multiplex techniques (e.g., microarrays) can produce potentially misleading results [16,17]. Multiple plausible explanations exist, such as small sample size, multitude of investigated genes, and polymorphisms of the target and the probe [18,19,20]. Thus, our study supports the necessity to confirm array data by qPCR. In the present paper we discuss only the miRNAs detected by the miRNA array and verified by qPCR.

In order to find possible pathophysiological/molecular pathways influenced by the observed miRNome changes, we searched our proteomic data acquired by mass spectrometric analysis of the same samples. We searched for both validated and predicted protein targets of the miRNAs, which were regulated in the opposite direction as the miRNAs in the same samples.

An important novel finding of this study is that miR-762 was the most upregulated miRNA in the EP group at the time of the peak miRNome response: at 6 h according to both miRNA microarray analysis and qPCR validation. MiR-762 was identified in remote ischemic preconditioning (rIPC) in mouse CD34+ bone marrow cells supporting its role in preconditioning [21]. However, the present study is the first demonstration of a possible role of miR-762 in LPS-induced AKI and preconditioning of the kidney. Mass spectrometry analysis of the renal proteome after LPS administration revealed that a predicted target of miR-762, the secretion associated Ras related GTPase 1B (Sar1b) may have been regulated by miR-762. Sar1b belongs to the Sar1-ADP ribosylation factor family of small GTPases [22], which govern the intracellular trafficking of proteins in protein-coated vesicles. Sar1b has been associated previously with oxidative stress and inflammation [23]. Here we describe for the first time a possible role of Sar1b in septic AKI. However, further possible targets of miR-762, such as IRFs and TLRs were not detected on our MS possibly due to the limitations of the total proteome analysis.

MiR-144 and miR-451 are closely clustered, evolutionally conserved bicistronic miRNA genes [24,25]. Our qPCR data showed similar expression pattern for these two miRNAs, supporting their close relationship. Li et al. revealed a protective role for miR-144-3p in remote (limb ischemia) preconditioning of the heart [26]. MiR-451a was reported to increase in RAW267.4 cells after LPS administration [27]. However, there is no previous report on the role of miR-144/451 cluster in the LPS-induced septic AKI.

Mass spectrometry analysis of the same renal samples revealed that the concentration of apuaporin-1 (Aqp1), an already validated target of miR-144-3p [28] decreased at 24 h when miR-144-3p was upregulated, suggesting a possible regulation of Aqp1 by miR-144-3p. LPS downregulated both mRNA and protein expression of Aqp1 in mouse kidneys [29,30]. Just as in our study, Aqp1 protein was first elevated and later decreased in the kidney [30]. Aqp1 mRNA was downregulated after renal ischemia-reperfusion injury as well [31] and urinary exosomal release of AQP1 and AQP2 was reduced in ischemic AKI [32]. Furthermore, Aqp1 knockout mice suffered more severe kidney injury after LPS administration [33] and Aqp1 overexpression was protective in LPS-challenged HK-2 cells [34]. Another transporter, solute carrier family 22 member 8 (Slc22a8), also known as the organic anion transporter 3 (Oat3), a predicted target of miR-144-3p was inversely regulated with miR-144-3p too. Sepsis and ischemia has been demonstrated to downregulate renal tubular function, including anion excretion [35]. Two previous studies also demonstrated significant downregulation of Slc22a8 (Oat3) already 6 h after LPS administration [36] time and dose dependently [35]. Taken together our data support a pathogenic role of Aqp-1 and Slc22a8 in LPS-induced AKI and raise the possibility that they are regulated by miR-144-3p.

Our data strongly support a role for miR-21 in the LPS-induced AKI, as according to the microarray it was one of the most upregulated miRNAs, and the 3p arm of miR-21 was upregulated at all time points after LPS administration. It has been described that miR-21 has a role in delayed ischemic preconditioning as its knock-down at the time of preconditioning exacerbated subsequent ischemia-reperfusion injury [37]. Several reports indicated that miR-21a modulated mainly the adaptive immune system, which effect could explain why its expression level was elevated at later time points in our experiment. We also observed in a previous study that miR-21 elevation was a late event—initiated first 24 h after an ischemic renal insult [38]. The immune-regulatory roles as well as the possible effect of miR-21 in mediating preconditioning are well characterized [39,40]. Finally, miR-21 has been reported to play a role in the LPS-induced renal injury [14]. The present study confirms these previously reported roles of miR-21.

Our study also supports the role of miR-146a-5p in septic AKI as it was markedly upregulated by LPS. MiR-146a is a regulator of pro-inflammatory cytokine production in macrophages upon LPS stimulation and may attenuate the immune response in monocytes [41]. MiR-146a-5p has been reported to play a role in LPS-induced septic AKI. It was also found to be essential in the LPS-induced cross-tolerance to renal ischemia by activating NF-kB signaling [6,42].

Mir-223-3p expression was significantly elevated at 6 and 24 h after LPS on the micro-array and at 1.5 and 24 h as shown by qPCR. Mir-223 is involved in hematopoiesis [43], and it has been associated with inflammatory disease states such as sepsis [44] and hepatic ischemia [45]. Wang et al. [46] reported that miR-223 downregulated the inflammatory response to LPS in RAW 264.7 murine macrophages, via suppressing the NF-κB signaling pathway. Thus, miR-223 may contribute to the immune-paralysis observed in LPS preconditioning. Furthermore, miR-223 had a model specific role in the kidney during experimental sepsis [10]. Finally, miR-223 participated in cecal ligation and puncture-induced preconditioning of the heart [11]. Our data support the involvement of miR-223-3p in LPS-induced AKI. The target search for miR-223 revealed that Slc4a4, the electrogenic sodium bicarbonate cotransporter 1 (NBCe1) was regulated inversely to miR-223-3p and miR-144-3p (Figure 5g,h). Thus, it is possible that Slc4a4 was downregulated by miR-223 and miR-144 in the late phase after LPS. The involvement of Slc4a4 in septic conditions has not been described before. However, miR-223-3p was found to promote cell proliferation and metastasis in clear cell renal cell carcinoma by downregulating SLC4A4 [47], further supporting the association of Slc4a4 and miR-223-3p.

In conclusion, we have described the renal miRNA expression changes and compared with mass spectrometric protein profile of the same kidney samples in response to LPS administration in a time-dependent manner. The miRNome changes were mild at 1.5 h, despite an already severe functional impairment. Primarily miRNA expression elevation dominated at 6 and 24 h, and these miRNAs started to decline by 48 h when the kidney also started to recover. One exception was miR-21-5p, which started to increase at 24 and remained elevated at 48 h. Besides miRNAs known to be altered by endotoxin administration, we found that miR-762 expression increased massively during the early phase of septic AKI. We also demonstrated that the miR-144/451 cluster was upregulated at 24 h and simultaneously, aquaporin-1, a validated target of miR-144, was downregulated. The miRNAs identified in this study might attenuate the LPS-induced immune response and may have an important role in renal inflammation.

## 4. Methods

### 4.1. Mice

We used 5–6-week-old male Naval Medical Research Institute (NMRI) mice, weighing 25–30 g (Toxi-Coop, Budapest, Hungary). The animals were housed under standard conditions with free access to standard rodent chow (Akronom Kft., Budapest, Hungary) and tap water ad libitum. The protocol was approved by the Animal Ethics Committee of Semmelweis University (No: XIV-1-001/2013-4/2012, XIV-1-001/2101-4/2012, XIV-1-001/2104-4/2012).

Other results from the same set of experiments have been previously published [48].

### 4.2. Endotoxin Preparations and Injection

LPS, prepared from *Escherichia coli* (*E. coli)* (0111:B4, Sigma-Aldrich, Budapest, Hungary), was suspended in sterile saline immediately before administration. Mice were divided into six groups:

Early phase (EP): 40 mg/kg LPS was administered i.p. in order to investigate the maximal molecular responses and the animals were sacrificed at 1.5 (n = 7) or 6 h (n = 7) after administration.

Late phase (LP): 10 mg/kg LPS was administered i.p. in order to investigate late molecular responses and the animals were sacrificed at 24 (n = 7) or 48 h (n = 7) after administration.

Control groups: equal volume of saline was administered i.p. in both the EP and LP settings (n = 7) to serve as negative controls.

Two animals died overnight, one in the 24-h and one in the 48-h group.

### 4.3. Organ Harvest

Mice were anticoagulated with 10 mL/kg (550 IU/mL) heparin (Ratiopharm, Ulm, Germany), i.p. 2 min prior to cervical dislocation. Blood was drawn from the transected vena cava and centrifuged for 2 min at 6000× *g* to obtain plasma samples. Mice were then perfused with 10 mL ice cold physiological saline through the left ventricle. The left kidney was removed and processed for RNA isolation.

### 4.4. Plasma Urea Determination

Plasma urea concentration was measured using a urease and glutamate-dehydrogenase enzymatic assay with colorimetric detection according to the manufacturer’s protocol (Diagnosticum Zrt., Budapest, Hungary). Briefly, the urease and glutamate-dehydrogenase containing reagent was added to the plasma samples. The conversion of NADH into NAD was measured photometrically at 340 nm. The urea concentration of the samples was calculated using a standard curve.

### 4.5. Total RNA Extraction and mRNA Real-Time PCR

Total RNA was isolated using TRI Reagent^®^ (Molecular Research Center, Inc., Cincinnati, OH, USA) according to the manufacturer’s protocol. Briefly, after phase separation with chloroform the supernatants were aspirated and mixed with isopropyl alcohol for RNA precipitation. The RNA pellets were washed twice with 70% ethyl alcohol and dissolved in RNase-free water (AccuGENE^TM^ Molecular Biology Water, Lonza, Basel, Switzerland). Concentrations and purity were quantified using Nanodrop 2000c Spectrophotometer (Thermo Fisher Scientific, Wilmington, DC, USA). RNA integrity was verified by electrophoretic separation on 1% agarose gel.

Reverse transcription of 1 μg total RNA into cDNA was carried out using random hexamer primers and the High-Capacity cDNA Archive Kit (Applied Biosystem, Foster City, CA, USA) according to the manufacturer’s protocol. Messenger RNA levels of TNF-α, IL-6, Lcn-2 (Table 3) were measured by real-time PCR using SensiFast SYBRGreen No-Rox kit (Bioline Reagents Ltd., London, UK) according to the manufacturer’s protocol. GAPDH was used as endogenous reference.

### 4.6. MicroRNA Microarray Profiling

We selected four samples from each group based on the TNF-α, IL-6, and Lcn-2 mRNA levels. The samples were selected in such a way that the mean and SD of the selected values were similar to the values measured in the whole groups.

Microarray measurements were conducted at Exiqon A/S (Vedbæk, Denmark). In brief, the quality of the total RNA was verified by an Agilent 2100 Bioanalyzer profile. Total RNA (750 ng) from both the test and reference (pooled) samples were labelled with Hy3™ and Hy5™ fluorescent label, respectively, using the miRCURY LNA™ microRNA Hi-Power Labeling Kit, Hy3™/Hy5™ (Exiqon, Denmark). The hybridization was performed according to the miRCURY LNA™ microRNA Array Instruction manual using a Tecan HS4800™ hybridization station (Tecan, Austria). After hybridization the miRCURY LNA™ microRNA Array slides were scanned using the Agilent G2565BA Microarray Scanner System (Agilent Technologies, Inc., USA). The image analysis was carried out using the ImaGene^®^ 9 (miRCURY LNA™ microRNA Array Analysis Software, Exiqon, Denmark). The quantified signals were background corrected (Normexp with offset value 10) and normalized using the global Lowess (Locally Weighted Scatterplot Smoothing) regression algorithm. Microarray data discussed in this publication have been deposited in NCBI’s Gene Expression Omnibus [49] and are accessible through GEO Series accession number GSE139919 (https://www.ncbi.nlm.nih.gov/geo/query/acc.cgi?acc=GSE139919).

### 4.7. MicroRNA Real-Time PCR

We performed real-time PCR in all samples to validate the microarray results. Samples were transcribed to cDNA using the Applied Biosystem™ TaqMan™ Advanced miRNA cDNA Synthesis Kit (Applied Biosystem, Foster City, CA, USA). The miRNA expression levels were measured with TaqMan™ Advanced miRNA Assay (Applied Biosystem, Foster City, CA, USA). Relative expressions were calculated using the 2^−ΔΔ^*^C^*^t^ method. Let-7g-5p miRNA was chosen as normalizing miRNA (stability value 0.057), using NormFinder software (MOMA—Department of Molecular Medicine, Aarhus, Denmark) [50].

### 4.8. Mass Spectrometry

Four kidney samples selected as most representative from each treatment group and four control kidney samples were processed for mass spectrometry analysis as described before [48]. LC-MS/MS analysis was performed with an EASY-nanoLC II HPLC unit (Thermo Fisher Scientific) coupled to an Orbitrap LTQ Velos mass spectrometer (Thermo Fisher Scientific) [48].

### 4.9. Data Analysis

Data analysis was performed as described previously [48], using MaxQuant proteomics software (version 1.6.0.13) for database search and quantification by spectral counting [51]. Raw data and database search files are available via ProteomeXchange with identifier PXD014664 [52]. Relative quantification of identified proteins was performed by label-free quantification (LFQ) algorithm in MaxQuant. Mass spectrometry results were checked for proteins previously identified with strong evidence as targets of all the significantly dysregulated miRNAs (based on MirTarbase [53] and literature search).

### 4.10. MicroRNA Target Prediction

MirDB [54,55] and microRNA.org [56] were searched for predicted targets of miRNAs. Mass spectrometry results were checked for predicted miRNA targets.

### 4.11. Statistics

ROUT method [57] (significance at the level *p* = 0.01) was performed to identify possible outliers, which were omitted from the analysis. In the miRNA microarray study, significantly changed miRNAs with a fold change above 1.5 or below 0.75 were considered differentially expressed. Only miRNAs with an average Hy3 signal intensity above 6.0 were included in the analysis. Messenger RNA and miRNA fold changes (based on qPCR assays) were calculated by dividing each normalized expression value with the mean of the respective control group. Logarithmic transformation of data was performed in case of significant inhomogeneity of variances indicated by Bartlett’s test. LFQ intensity values of proteins were log2 transformed for statistical analysis. Statistical analysis was performed using one-way ANOVA. For multiple comparisons Dunnett’s post hoc test was used. GraphPad Prism (version 6.01, GraphPad Software Inc, San Diego, CA, USA) was used for all statistical analyses and creation of graphs.

## Figures and Tables

**Table 1:** The members of the three clusters based on the two-way hierarchical clustering. Cluster I: miRNAs jointly upregulated in EP groups at 6 h, Cluster II: miRNAs jointly upregulated in LP groups, Cluster III: miRNAs jointly downregulated in LP groups. Grey highlight: miRNAs identified also by the analysis of ranking.

Cluster I (EP: 6 h)	Cluster II (LP)	Cluster III (LP)
miR-21a-3p	miR-20a-5p	miR-34c-3p
miR-204-3p	miR-21a-5p	miR-150-5p
miR-291a-3p	miR-144-3p	miR-1839-3p
miR-361-3p	miR-146a-5p	
miR-466n-3p	miR-155	
miR-665-3p	miR-451a	
miR-711		
miR-762		
miR-763		
miR-1934-5p		
miR-1971		
miR-2137		
miR-2861		
miR-3090-5p		
miR-3100-3p		
miR-3102-5p		
miR-5116		
miR-5126		
miR-5129-5p		
miR-5626-3p		
miR-5627-3p		

**Table 2:** Fold changes in miRNAs differentially expressed.

miRNA	1.5 h	6 h	24 h	48 h
miR-204-3p	2.82 ± 0.20 ***	1.88 ± 0.28 ***	1.08 ± 0.10 (ns)	1.21 ± 0.25 (ns)
miR-3102-5p	1.11 ± 0.23 (ns)	2.77 ± 0.52 ***	1.05 ± 0.32 (ns)	1.15 ± 0.25 (ns)
miR-762	1.19 ± 0.27 (ns)	2.69 ± 0.34 ***	1.16 ± 0.32 (ns)	1.24 ± 0.22 (ns)
miR-2137	0.96 ± 0.01 (ns)	2.61 ± 0.26 ***	1.41 ± 0.13 (ns)	1.42 ± 0.63 (ns)
miR-3090-5p	1.21 ± 0.16 (ns)	2.50 ± 0.61 *	1.11 ± 0.35 (ns)	1.36 ± 0.42 (ns)
**miR-2861**	1.06 ± 0.29 (ns)	2.46 ± 0.40 **	2.22 ± 0.72 **	1.49 ± 0.48 (ns)
miR-665-3p	1.25 ± 0.22 (ns)	2.32 ± 0.22 ***	0.93 ± 0.13 (ns)	0.98 ± 0.11 (ns)
miR-5129-5p	1.17 ± 0.34 (ns)	2.25 ± 0.69 *	0.94 ± 0.37 (ns)	0.95 ± 0.15 (ns)
**miR-21a-3p**	1.36 ± 0.12 (ns)	2.00 ± 0.20 ***	1.88 ± 0.18 ***	1.34 ± 0.17 (ns)
miR-5116	1.05 ± 0.07 (ns)	1.76 ± 0.17 ***	1.20 ± 0.18 (ns)	1.00 ± 0.15 (ns)
miR-3100-3p	1.03 ± 0.08 (ns)	1.67 ± 0.18 ***	1.28 ± 0.22 (ns)	1.10 ± 0.11 (ns)
miR-711	1.00 ± 0.08 (ns)	1.66 ± 0.24 **	1.23 ± 0.08 (ns)	1.31 ± 0.47 (ns)
miR-466n-3p	1.01 ± 0.06 (ns)	1.61 ± 0.25 *	1.02 ± 0.20 (ns)	0.90 ± 0.08 (ns)
miR-223-3p	1.40 ± 0.20 (ns)	1.60 ± 0.10 **	1.33 ± 0.12 *	1.23 ± 0.16 (ns)
miR-3474	1.01 ± 0.01 (ns)	1.60 ± 0.24 **	1.12 ± 0.09 (ns)	1.02 ± 0.13 (ns)
miR-21a-5p	1.07 ± 0.2 (ns)	1.39 ± 0.18 *	4.44 ± 0.66 ***	4.59 ± 1.34 **
miR-451a	1.63 ± 0.98 (ns)	1.08 ± 0.33 (ns)	3.76 ± 1.56 **	2.22 ± 2.41 (ns)
miR-144-3p	1.37 ± 0.87 (ns)	1.10 ± 0.24 (ns)	2.56 ± 1.04 **	1.65 ± 1.43 (ns)
**miR-2861**	1.06 ± 0.29 (ns)	2.46 ± 0.40 **	2.22 ± 0.72 **	1.49 ± 0.48 (ns)
**miR-21a-3p**	1.36 ± 0.12 (ns)	2.00 ± 0.20 ***	1.88 ± 0.18 ***	1.34 ± 0.17 (ns)
miR-146a-5p	1.10 ± 0.08 (ns)	0.98 ± 0.08 (ns)	1.51 ± 0.14 **	1.58 ± 0.21 **
miR-1839-3p	1.02 ± 0.21 (ns)	0.92 ± 0.24 (ns)	0.57 ± 0.09 **	0.74 ± 0.16 **
miR-34c-3p	0.96 ± 0.19 (ns)	0.87 ± 0.24 (ns)	0.57 ± 0.17 **	0.68 ± 0.16 **
miR-150-5p	1.01 ± 0.20 (ns)	0.79 ± 0.13 (ns)	0.59 ± 0.06 ***	0.63 ± 0.13 ***
miR-129-1-3p	0.90 ± 0.08 (ns)	0.81 ± 0.06 *	0.70 ± 0.05 ***	0.65 ± 0.10 ***
miR-34b-3p	1.01 ± 0.11 (ns)	0.85 ± 0.16 (ns)	0.74 ± 0.04 **	0.81 ± 0.12 (ns)
miR-3070a-5p/ miR-3070b-5p	1.05 ± 0.11 (ns)	0.85 ± 0.09 *	0.78 ± 0.08 (ns)	0.71 ± 0.07 **

Fold changes of miRNA expression, differentially expressed after LPS administration compared to the saline group measured by microarray. (>1 values: LPS-induced upregulation <1: reduced expression: above and below the line, respectively). Underlined letters: qPCR validated miRNAs. Bold letters: miRNAs regulated similarly in both EP and LP (miR-21a-3p, miR-2861). Grey highlight: miRNAs identified also by the heat map cluster analysis. One-way ANOVA; *: *p* < 0.05, **: *p* < 0.01, ***: *p* < 0.001, ns: not significant. EP (1.5 and 6 h) and LP (24 and 48 h) data were statistically analyzed separately (double line).

**Table 3:** Applied primer sequences for qPCR.

Target Gene	Forward Primer	Reverse Primer
TNF-α	AAATGGCCTCCCTCTCATCA	AGATAGCAAATCGGCTGACG
IL-6	CAAAGCCAGAGTCCTTCAGAGA	GGTCTTGGTCCTTAGCCACTC
Lcn-2	ACGGACTACAACCAGTTCGC	AATGCATTGGTCGGTGGGG
GAPDH	TTCACCACCATGGAGAGGGC	GGCATGGACTGTGGTCATGA
